# Synthesis of the Ca^2+^-mobilizing messengers NAADP and cADPR by intracellular CD38 enzyme in the mouse heart: Role in β-adrenoceptor signaling

**DOI:** 10.1074/jbc.M117.789347

**Published:** 2017-05-24

**Authors:** Wee K. Lin, Emma L. Bolton, Wilian A. Cortopassi, Yanwen Wang, Fiona O'Brien, Matylda Maciejewska, Matthew P. Jacobson, Clive Garnham, Margarida Ruas, John Parrington, Ming Lei, Rebecca Sitsapesan, Antony Galione, Derek A. Terrar

**Affiliations:** From the ‡ Department of Pharmacology, University of Oxford, Mansfield Road, Oxford OX1 3QT, United Kingdom,; the § Department of Chemistry, Chemistry Research Laboratory, University of Oxford, Mansfield Road, Oxford OX1 3TA, United Kingdom,; the ¶ Department of Pharmaceutical Chemistry, University of California, San Francisco, California 94158, and; the ‖ Faculty of Biology, Medicine, and Health, University of Manchester, Manchester M13 9NT, United Kingdom

**Keywords:** cardiac hypertrophy, CD38, cyclic ADP-ribose (cADPR), heart, nicotinic acid adenine dinucleotide phosphate (NAADP), sarcoplasmic reticulum (SR), Ca^2+^, β-adrenoceptor, cardiac arrhythmia, lysosomes

## Abstract

Nicotinic acid adenine dinucleotide phosphate (NAADP) and cyclic ADP-ribose (cADPR) are Ca^2+^-mobilizing messengers important for modulating cardiac excitation–contraction coupling and pathophysiology. CD38, which belongs to the ADP-ribosyl cyclase family, catalyzes synthesis of both NAADP and cADPR *in vitro*. However, it remains unclear whether this is the main enzyme for their production under physiological conditions. Here we show that membrane fractions from WT but not *CD38*^−/−^ mouse hearts supported NAADP and cADPR synthesis. Membrane permeabilization of cardiac myocytes with saponin and/or Triton X-100 increased NAADP synthesis, indicating that intracellular CD38 contributes to NAADP production. The permeabilization also permitted immunostaining of CD38, with a striated pattern in WT myocytes, whereas *CD38*^−/−^ myocytes and nonpermeabilized WT myocytes showed little or no staining, without striation. A component of β-adrenoreceptor signaling in the heart involves NAADP and lysosomes. Accordingly, in the presence of isoproterenol, Ca^2+^ transients and contraction amplitudes were smaller in *CD38*^−/−^ myocytes than in the WT. In addition, suppressing lysosomal function with bafilomycin A1 reduced the isoproterenol-induced increase in Ca^2+^ transients in cardiac myocytes from WT but not *CD38*^−/−^ mice. Whole hearts isolated from *CD38*^−/−^ mice and exposed to isoproterenol showed reduced arrhythmias. SAN4825, an ADP-ribosyl cyclase inhibitor that reduces cADPR and NAADP synthesis in mouse membrane fractions, was shown to bind to CD38 in docking simulations and reduced the isoproterenol-induced arrhythmias in WT hearts. These observations support generation of NAADP and cADPR by intracellular CD38, which contributes to effects of β-adrenoreceptor stimulation to increase both Ca^2+^ transients and the tendency to disturb heart rhythm.

## Introduction

ADP-ribosyl cyclases (ARCs)^5^ are enzymes capable of converting NAD into cyclic adenosine diphosphate ribose (cADPR). In addition, some of these cyclases (for example, CD38 and *Aplysia* cyclase), can (at least under *in vitro* conditions) also produce nicotinic acid adenine dinucleotide phosphate (NAADP) using NADP as substrate in the presence of nicotinic acid (NA) (see reviews in Ref. 1). cADPR and NAADP are both potent Ca^2+^-releasing messengers (2, 3) that play significant roles in various signaling pathways in plants and animals, including humans (see reviews in Refs. 4, 5). Cellular functions mediated by these two messengers include hormonal secretion, smooth muscle contraction, immune responses, fertilization, and neuromodulation (4, 5). In the heart, both cADPR and NAADP modulate excitation–contraction coupling (6–9). These effects seem particularly important after stimulation of β-adrenoceptors, presumably associated with the elevated levels of NAADP and cADPR (7, 10, 11). It has been postulated that the increased concentrations of these messengers result from enhanced production by endogenous enzymes and that excessive production of these molecules contributes to the development of cardiac hypertrophy and arrhythmias associated with stimulation of β-adrenoceptors (9, 12, 13). The enzyme(s) responsible for the production of cADPR and NAADP is/are therefore a potential therapeutic target, and drugs, including SAN4825, to target these enzyme(s) are under development (14). However, the nature and location of ARCs and/or NAADP-synthesizing enzymes in the heart remain unclear.

It was mentioned above that CD38 can function as an ARC catalyzing the synthesis of both cADPR and NAADP. However, a recent study on cardiac tissue suggests that CD38 mediates the synthesis of cADPR but not NAADP (15). This paper proposes that another cardiac enzyme on lysosomes synthesizes NAADP in response to intracellular Ca^2+^ fluxes in the myocyte following stimulation of β-adrenoceptors. The enzyme was referred to as NAADP-synthesizing enzyme. A further proposal was that NAADP produced within the lysosome causes Ca^2+^ release from this organelle and that this Ca^2+^ promotes cADPR synthesis by CD38 in neighboring endosomes. cADPR transported out of the endosome was then proposed to regulate ryanodine receptor opening in the sarcoplasmic reticulum (SR). Suppression of CD38 expression in *CD38*^−/−^ mice was shown to reduce the cardiac hypertrophy that is associated with chronic exposure to the β-adrenoreceptor agonist isoproterenol (15). The proposal that CD38 promotes cADPR synthesis within the organelle is consistent with the commonly accepted view that CD38 can show activity as an ectoenzyme at the plasma membrane with an active site facing away from the cytosol (16). Work on lymphocytes first established the external orientation of CD38 (17). Because targets for NAADP and cADPR in cardiac muscle appear to be intracellular, the physiological relevance in the heart of enzyme activity of an ecto-CD38 remains unclear. Some have proposed expression of intraorganellar ARC (with an active site facing the lumen) in other tissues for cADPR and NAADP synthesis (see reviews in Refs. 18, 19). Evidence from sea urchin egg supports the synthesis of cADPR within organelles, followed by transport of the active substance into the cytosol to enable its participation in Ca^2+^ signaling (20). However, it has recently been suggested that CD38 can also exist in the opposite orientation, with the active site of the enzyme facing the cytosol, and that the active site can still function to promote synthesis of cADPR in this topological orientation (21–23).

The aim of this study was to further characterize the nature and identity of cADPR- and NAADP-synthesizing enzymes in cardiac muscle, exploring their possible location on intracellular membranes and investigating the hypothesis that CD38 might function as the main enzyme responsible for the synthesis for both Ca^2+^-mobilizing messengers. The contributions of this pathway to physiological excitation–contraction coupling and pathological development of disturbances of cardiac rhythm were also investigated because this pathway provides a novel target for the development of antiarrhythmic drugs.

## Results

### CD38 is the main enzyme responsible for cardiac synthesis of NAADP and cADPR

**Figure 1.**
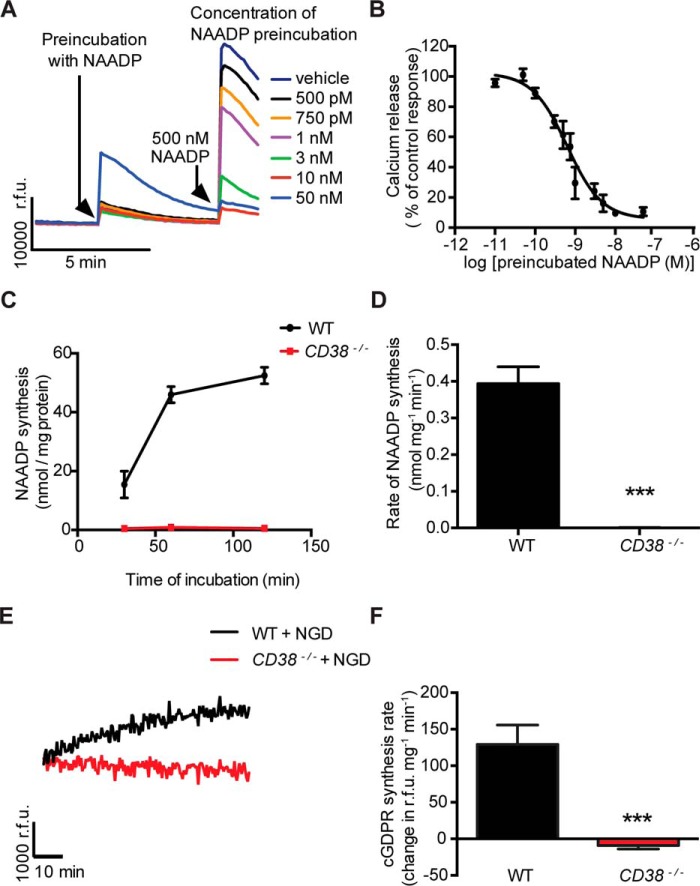
***In vitro* ADP-ribosyl cyclase activities and NAADP production were not detected in mixed-membrane preparations from *CD38*^−/−^ mouse heart.** We evaluated the ability of a membrane preparation containing plasmalemma and SR from WT but not *CD38*^−/−^ mouse heart to catalyze the synthesis of NAADP and cADPR. *A*, example traces of the NAADP measurement assay system based on SUEH, in which increasing concentrations of NAADP show a desensitization response to the activating substance NAADP. *B*, observations reflecting enhanced desensitization at increasing concentrations of NAADP were used to construct the calibration curve. *C*, using this assay, we show that a membrane preparation from WT mice catalyzed the synthesis of NAADP, whereas a similar preparation from *CD38*^−/−^ mouse heart lacked this ability. The time course of NAADP production is shown. *D*, the average rate of NAADP synthesis from these data (*n* = 4 for both groups). *E* and *F*, the membrane preparation from WT mice catalyzed synthesis of cGDPR (an analogue of cADPR) with NGD as a substrate (the time course is shown in *E*, and a bar graph showing the average rate of cGDPR synthesis is shown in *F*), whereas membranes from *CD38*^−/−^ mice lacked this ability (n = 6 for both groups). *r.f.u.* indicates relative florescence unit. Note that WT membranes showed clear synthesis of both messengers, whereas there was no detectable synthesis with *CD38*^−/−^ membranes. Data are expressed as mean ± S.E. ***, *p* ≤ 0.001; *n* = number of preparations.

A membrane-enriched preparation containing sarcolemma and SR was derived from mouse heart muscle both from WT and *CD38*^−/−^ mice. A sensitive bioassay for measuring NAADP was provided by sea urchin egg homogenates (SUEHs) (24), which show a homologous desensitization of the Ca^2+^-releasing effect of NAADP at remarkably low concentrations (from ∼10^−10^ to 10^−8^ m). Example traces and a calibration curve are shown in Fig. 1, *A* and *B*. The membrane-enriched preparation from WT hearts produced NAADP when supplied with NADP and NA (Fig. 1, *C* and *D*). Interestingly, there was no comparable synthesis of NAADP with mixed membranes from *CD38*^−/−^ hearts (*n* = 3; *p* ≤ 0.001; Fig. 1, *C* and *D*). A widely accepted conventional nicotinamide guanine dinucleotide (NGD) assay was used to determine the rate of cADPR synthesis (25). Using this assay, we detected the cyclization of NGD (an analogue of NAD) into cyclic GDP-ribose (cGDPR, a fluorescent analogue of cADPR, by membranes from WT hearts but not from *CD38*^−/−^ hearts (*n* = 6; *p* ≤ 0.001; Fig. 1, *E* and *F*). These observations are consistent with the hypothesis that CD38 is the main enzyme responsible for the cardiac synthesis of both NAADP and cADPR.

### Intracellular location of CD38 and possible association with the SR

**Figure 2.**
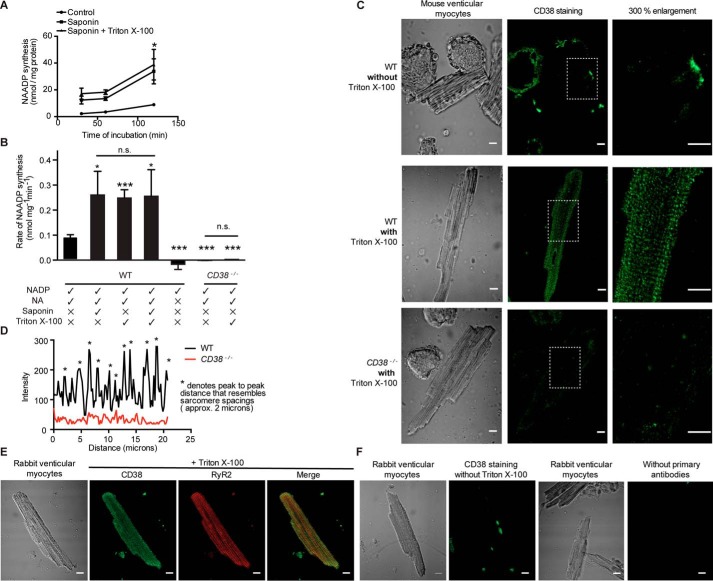
**Permeabilization of cardiac myocytes with Triton X-100 and/or saponin enhanced NAADP production and permitted immunolabeling of CD38.** *A*, the rate of NAADP synthesis was higher after permeabilization of the cell membranes with saponin alone, and permeabilization with Triton X-100 in addition to saponin did not further increase the rate of NAADP synthesis (*n* = 3 in each group). *B*, this point is further illustrated in the bar graph, which also shows that the ability to synthesize NAADP was lost in myocytes from *CD38*^−/−^ mice (*n* = 4). Omission of NA also abolished synthesis of NAADP in intact cells (*n* = 5). *C*, immunolabeling of CD38 using rabbit anti-human CD38 antibody without Triton X-100 showed little staining, although there were surface patches with higher fluorescence intensity (*top panels*). Following membrane permeabilization with Triton X-100 to allow access of the antibody to the cell interior, there was clear staining with a striated pattern in WT (*center panels*) but not in permeabilized *CD38*^−/−^ cardiac myocytes (*bottom panels*). *D*, the representative intensity–distance plot (*bottom panel*) shows that, in permeabilized cells, the staining observed in the WT myocyte had a much higher intensity than in the *CD38*^−/−^ myocyte and showed multiple peaks with a repeating distance interval that resembled the sarcomere spacing. *E*, similar observations in rabbit ventricular myocytes. The fluorescent images of myocytes permeabilized with Triton X-100 showed clear labeling with CD38 antibody. There was a striated pattern with a similar spacing as that shown by immunolabeling of RyR2. *F*, no labeling with a striated pattern was observed when Triton X-100 or primary antibodies were omitted. The images show representative staining of the major observation (≥75%) in each group (*n* ≥ 20). *Scale bars* = 10 μm, *n* = number of cells. Data are expressed as mean ± S.E. *, *p* ≤ 0.05; ***, *p* ≤ 0.001; *n.s.*, not significant.

Fig. 2*A* shows that, when the enzymatic activity of intact cardiac ventricular myocytes from WT mice was investigated using the same assay procedures as for the membrane-enriched preparation from heart muscle above, there was very little synthesis of NAADP when cells were supplied with NADP and NA in the extracellular solution. However, permeabilization of the ventricular cell membranes with saponin (0.01% w/v) caused a substantial increase in the rate of production of NAADP, consistent with access of substrates to the cell interior of healthy cells, permitting production of NAADP by enzymes with intracellular active sites (Fig. 2*A*). As saponin preferentially targets cholesterol-rich membranes (26) such as the sarcolemma, rather than the SR, we used Triton X-100 (0.1% v/v) to further expose potential intracellular enzymes (perhaps including those with lumen-facing active sites in various organelles). However, Triton X-100 permeabilization in the presence of saponin failed to cause a substantial increase in the rate of NAADP production (Fig. 2*A*). There appeared to be little or no difference between the effects of the two membrane-permeabilizing agents applied alone or in combination (Fig. 2*B*). The above experiments were repeated on intact myocytes from *CD38*^−/−^ hearts, and there was little or no synthesis of NAADP both in intact myocytes and after membrane permeabilization with Triton X-100 (Fig. 2*B*). Control experiments showed that neither Triton X-100 nor saponin at the concentration used in these experiments affected the ability of the sea urchin egg homogenate bioassay to measure NAADP (supplemental Fig. S1).

A rabbit polyclonal antibody to human CD38 was used to further investigate the presence of this enzyme in mouse ventricular myocytes because the amino acid sequence (residues 1–170) used to generate the antibody showed extensive similarities between the two species (supplemental Fig. S2). When the plasma membrane was intact in the absence of Triton X-100, there was little immunolabeling in WT mouse cardiac myocytes, and this labeling appeared to be concentrated in membrane patches (Fig. 2*C*, *top panels*). When Triton X-100 was used to permeabilize the cell membrane, the labeling by the CD38 antibody was much more extensive and showed striations with a spacing corresponding to the sarcomere length of slightly less than 2 μm (Fig. 2*C*, *center panels*). Convincing evidence that the human CD38 antibody was indeed labeling the mouse CD38 was provided by the observation that little or no labeling was detected in permeabilized cardiac ventricular myocytes from *CD38*^−/−^ mice (Fig. 2*C*, *bottom panels*). This striated pattern with a spacing close to 2 μm in myocytes from WT but not *CD38*^−/−^ mice is also supported by Fig. 2*D*, which shows plots of intensity for a line positioned along the long axis of the cell. Labeling by the antibody to human CD38 was also investigated in ventricular myocytes isolated from rabbit heart. In the presence of Triton X-100 to permeabilize cell membranes, immunolabeling with a striated pattern was again observed (Fig. 2*E*) and appeared to be strikingly similar to that observed in WT mouse myocytes. Again, the separation between striations appeared to correspond to sarcomere length, and labeling of ryanodine receptors (RyR2) showed a similar spacing. This immunolabeling with a striated pattern was not observed when Triton X-100 or the primary antibody was omitted (Fig. 2*F*). It therefore appears that membrane permeabilization was necessary for maximal NAADP synthesis and for labeling of intracellular CD38 in ventricular myocytes from WT mice and that neither NAADP synthesis nor intracellular labeling of CD38 were observed in ventricular myocytes from *CD38*^−/−^ mice.

In addition, CD38 labeling on permeabilized rabbit atrial myocytes was also found to have a striated pattern, similar to that observed in both mouse and rabbit ventricular myocytes (supplemental Fig. S3). This observation provides support for the association of CD38 with the SR rather than t tubules (see “Discussion”).

The observation that CD38 appears to be associated with the SR prompted us to investigate enzyme activity in a sheep SR vesicle preparation that is commonly used to investigate heart SR proteins, including RyR2 (27–34). cGDPR synthesis with NGD as substrate was observed in this sheep cardiac SR preparation, consistent with the presence of ARC activity (supplemental Fig. S4*A*). As expected, the effects were reduced in the presence of a CD38 inhibitor, nicotinamide (35). In supplemental Fig. S4*B*, we show that isolated sheep cardiac SR vesicles were also able to synthesize NAADP when provided with NADP and NA. Both heavy (HSR) and light (LSR) SR fractions exhibited comparable NAADP synthesis (HSR, 1.24 ± 0.60 nmol mg^−1^ min^−1^; LSR, 1.309 ± 0.390 nmol mg^−1^ min^−1^; *n* = 4; *p* = 0.9226). This synthesis did not occur when the sheep cardiac SR was provided with NADP alone or NA alone (supplemental Fig. S4*C*). This observation in sheep cardiac SR vesicles is consistent with our hypothesis of cardiac CD38 being expressed intracellularly and preferentially on the SR.

### The effects of isoproterenol to increase the amplitudes of Ca^2+^ transients (CaTs) and contractions accompanying electrical stimulation were smaller in cardiac myoctes from CD38^−/−^ mice than in those from WT myocytes

**Figure 3.**
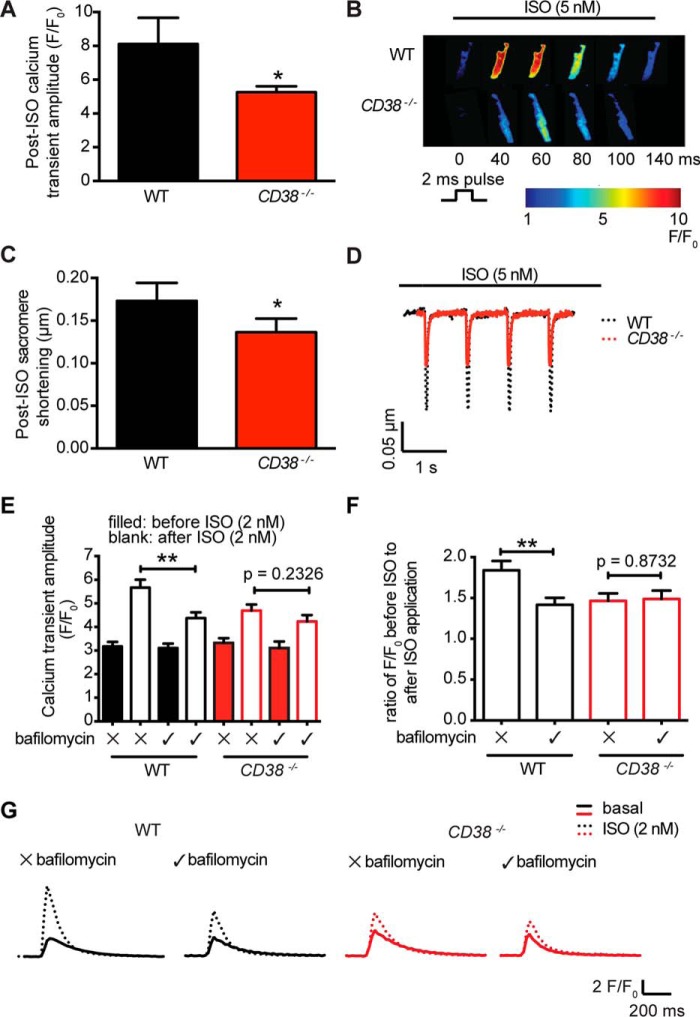
**Contribution of NAADP signaling pathway to the β-adrenoreceptor-mediated positive inotropy was lost in the *CD38*^−/−^ ventricular myocytes.** Ca^2+^ transients were triggered by cardiac action potentials in ventricular myocytes from WT and *CD38*^−/−^ mice. *A*, the amplitudes of Ca^2+^ transients in WT (*n* = 9) and *CD38*^−/−^ myocytes (*n* = 15) are shown in the presence of isoproterenol. *B*, example images. Under these conditions, amplitudes of CaTs accompanying action potentials were smaller in cardiac myocytes from *CD38*^−/−^ mice than from WT mice. *ISO*, isoproterenol. *C*, sarcomere shortening in the presence of isoproterenol accompanying action potentials was also smaller in myocytes from *CD38*^−/−^ mice than from WT mice (WT, *n* = 12; *CD38*^−/−^, *n* = 11). *D*, example traces. The observations support a functional role for the CD38 enzyme during β-adrenergic receptor stimulation. *E*, the effects of isoproterenol on the amplitude of CaTs with and without bafilomycin A1 (10 μm) in ventricular myocytes from WT and *CD38*^−/−^ mice (*n* = 16–25 in each group). *F*, these observations are replotted to show the magnitude of the isoproterenol-induced changes with and without bafilomycin A1 in myocytes from WT and *CD38*^−/−^ mice. *G*, representative traces. Bafilomycin A1 reduced the effect of isoproterenol on the amplitude of CaTs in myocytes from WT but not *CD38*^−/−^ mice. The isoproterenol-induced increase in amplitudes of CaTs was therefore less in the presence of bafilomycin A1 in WT myocytes, whereas, in myocytes from *CD38*^−/−^ mice, the isoproterenol-induced increases in CaT amplitude were similar with and without bafilomycin A1. Data are expressed as mean ± S.E. *, *p* ≤ 0.05; ** *p* ≤ 0.01; *n* = number of cells.

The functional effects of genetic knockout of CD38 were investigated in cardiac ventricular myocytes. Previous work has shown that levels of NAADP and cADPR are elevated following β-adrenoreceptor stimulation with isoproterenol (7, 11). It was also found that both NAADP and cADPR contribute, by different mechanisms, to the isoproterenol-induced increase in the amplitude of CaTs and contractions evoked by electrical stimulation in cardiac myocytes (7–9, 12). Consistent with these observations and the hypothesis that CD38 provides the major synthetic pathway for NAADP and cADPR, it was found that the amplitudes of CaTs and contractions in the presence of isoproterenol were smaller in cardiac myocytes from *CD38*^−/−^ mice than in myocytes from WT mice (Fig 3, *A–D*).

### Bafilomycin A1, to inhibit lysosomal NAADP function, reduced the increase in amplitude of CaTs caused by isoproterenol in electrically stimulated myocytes from WT but not CD38^−/−^ mice

In the case of NAADP actions, previous work has demonstrated that a component of the isoproterenol-induced increase in the amplitude of CaTs induced by electrical stimulation appears to depend on NAADP acting via TPC2 channels in lysosomal membranes (9). Lysosomes have also been shown to form associations with the SR, consistent with the formation of Ca^2+^ microdomains between the organellar membranes (36). In addition, it has been shown that, when lysosomal NAADP function is disrupted with bafilomycin A1, the increase in CaT amplitude caused by isoproterenol is reduced (7, 9). The reduction in the isoproterenol-induced increase in the amplitude of CaT following bafilomycin A1 treatment (10 μm) is shown again here for WT myocytes but, in contrast bafilomycin A1, failed to reduce the isoproterenol-induced increase in CaT amplitude in myocytes from *CD38*^−/−^ mice (Fig. 3*E*). This was also evident from the ratio of effects of isoproterenol with and without bafilomycin A1 (Fig. 3*F*, showing a lack of difference in *CD38*^−/−^ myocytes compared with a reduced effect in the WT). Sample records are shown in Fig. 3*G*. These observations are therefore consistent with the hypothesis that cardiac myocytes from *CD38*^−/−^ mice are unable to synthesize significant amounts of NAADP and that, therefore, this pathway can no longer be functionally suppressed by bafilomycin A1. The observations provide further support for a role for NAADP synthesized by CD38 in this physiological process in WT cardiac muscle. The remaining isoproterenol-induced increases in myocytes from *CD38*^−/−^ mice are likely to have resulted, at least in part, from the expected PKA-mediated effects on L-type Ca^2+^ channels and phospholamban that are thought not to involve CD38 (37). The CD38-dependent pathway underlying these observations is considered at greater length under “Discussion.”

### In vitro and in silico evidence showed that a novel inhibitor of cardiac ARC, SAN4825, also inhibited NAADP synthesis at physiological pH, possibly through targeting CD38

**Figure 4.**
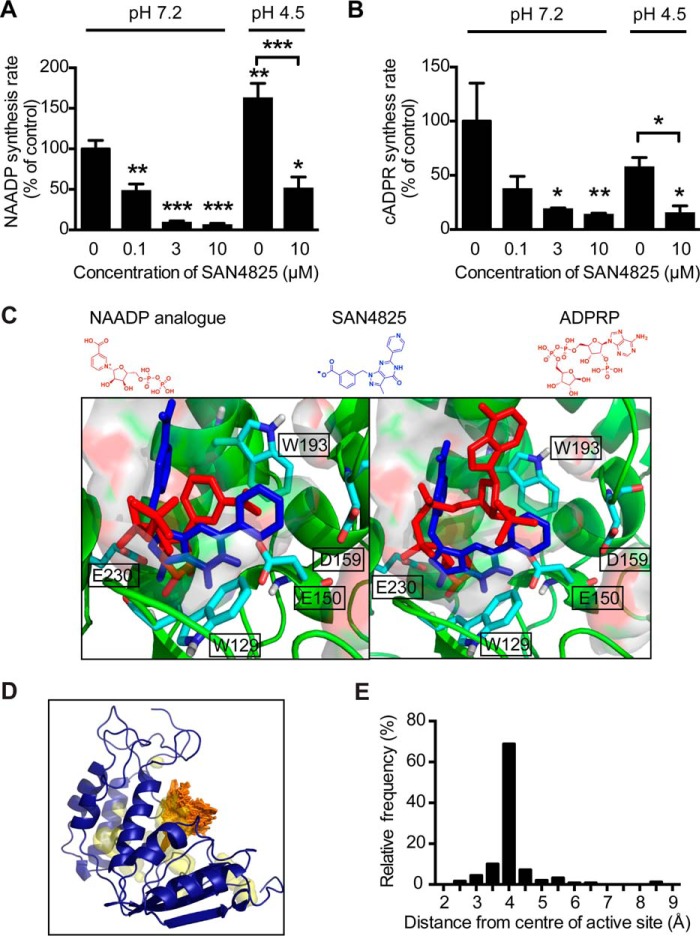
***In vitro* and *in silico* evidence for the action of SAN4825 to inhibit NAADP and cADPR production through targeting CD38.** *A* and *B*, SAN4825 inhibited NAADP (*A*) and cADPR (*B*) synthesis in mouse mixed-membrane preparations at both pH 7.2 and pH 4.5. Note that the synthesis of NAADP in the absence of SAN4825 was higher at pH 4.5 than at pH 7.2. *C*, superimposition of the binding conformation of the NAADP analogue in human CD38 (*left*, *red*, PDB code 4F46, chain B) on the most favorable binding conformation of SAN4825 (*left*, *blue*) in mouse CD38 (PDB code 2EG9) at pH 7.2 following Glide docking. A superimposition of the binding conformation of ADPRP in human CD38 (*right*, *red*, PDB code 4F46, chain A) on the most favorable binding conformation of SAN4825 (*right*, *blue*) in mouse CD38 (PDB code 2EG9) is also shown (*right*). *D*, the predicted binding sites (*orange*) for SAN4825 in mouse CD38 following blind docking in the SwissDock server. Inner cavities are shown in *yellow. E*, the distances between the predicted docking sites and the center of the active site were plotted. Data are expressed as mean ± S.E. *, *p* ≤ 0.05; **, *p* ≤ 0.01; ***, *p* ≤ 0.001; *n.s.*, not significant; *n* ≥ 3 preparations in each group.

SAN4825 is a novel drug developed as an inhibitor of cardiac-specific ARCs and has been shown (14) to suppress the ability of a rat SR membrane preparation to synthesize a fluorescent analogue of cADPR (IC_50_ ≈ 1.3 μm). This drug was also shown to partially inhibit the ARC activity of human purified CD38 at higher concentration (14). We tested the ability of SAN4825 to inhibit both NAADP and cADPR synthesis by the mouse membrane preparation for which the evidence reported above shows CD38 to be the major enzyme catalyzing synthesis of these Ca^2+^-mobilizing messengers. SAN4825 was observed to cause a concentration-dependent inhibition of both NAADP synthesis (Fig. 4*A*) and cADPR synthesis (Fig. 4*B*). A similar inhibition of NAADP synthesis by SAN4825 was also observed in saponin-permeabilized myocytes from mouse hearts (supplemental Fig. S5). SAN4825 inhibition of NAADP synthesis was observed to be greater at pH 7.2, and this will be considered at greater length under “Discussion.”

The active site residues Glu-150, Asp-159, Glu-230, Trp-129, and Trp-193 in mouse CD38 correspond to Glu-146, Glu-226, Asp-155, Trp-125, and Trp-189 in human CD38. These residues in human CD38 are believed to play an important role in NAADP and cADPR synthesis following mutagenesis studies (38). In a docking simulation using Glide, we demonstrate that the most favorable docking conformation of SAN4825 at pH 7.2 in mouse CD38 (PDB code 2EG9 (39)) is in close proximity to these active-site residues (Fig. 4*C*). Further superimposition of the binding conformation of SAN4825 in mouse CD38 with the binding conformations of an NAADP analogue and ADPRP in human CD38 (PDB code 4F46 (40)) suggests that SAN4825 occupies the same binding site as these substrates, in close proximity to Glu-230 of mouse CD38 (Fig. 4*C*). The pyridine ring of SAN4825 also showed π–π stacking with Trp-193 of mouse CD38. These π–π interactions were also observed in the crystal structure of wild-type human CD38 in complex with the NAADP analogue and ADPRP (40).

Further computational studies also demonstrated that the single active site is the only plausible binding site of SAN4825. Specifically, blind docking results using SwissDock showed that approximately 90% of the predicted docking sites (250 predictions in total) of SAN4825 in mouse CD38 are within 4.5 Å from the center of the active site (Fig. 4, *D* and *E*). SiteMap also presented a similar result, showing that the active site is the only site to present a SiteScore value closer to 1.0 (0.97), indicative of a difficult but druggable site with an estimated volume of 286 Å^3^. The other binding sites presented SiteScore values close to 0.8, suggestive of undruggable cavities with volumes smaller than 120 Å^3^. Taken together, these observations suggest that SAN4825 acts as a competitive inhibitor of NAADP and cADPR synthesis.

### CD38^−/−^ hearts were more resistant to arrhythmias during overstimulation of the β-adrenoreceptor pathway

**Figure 5.**
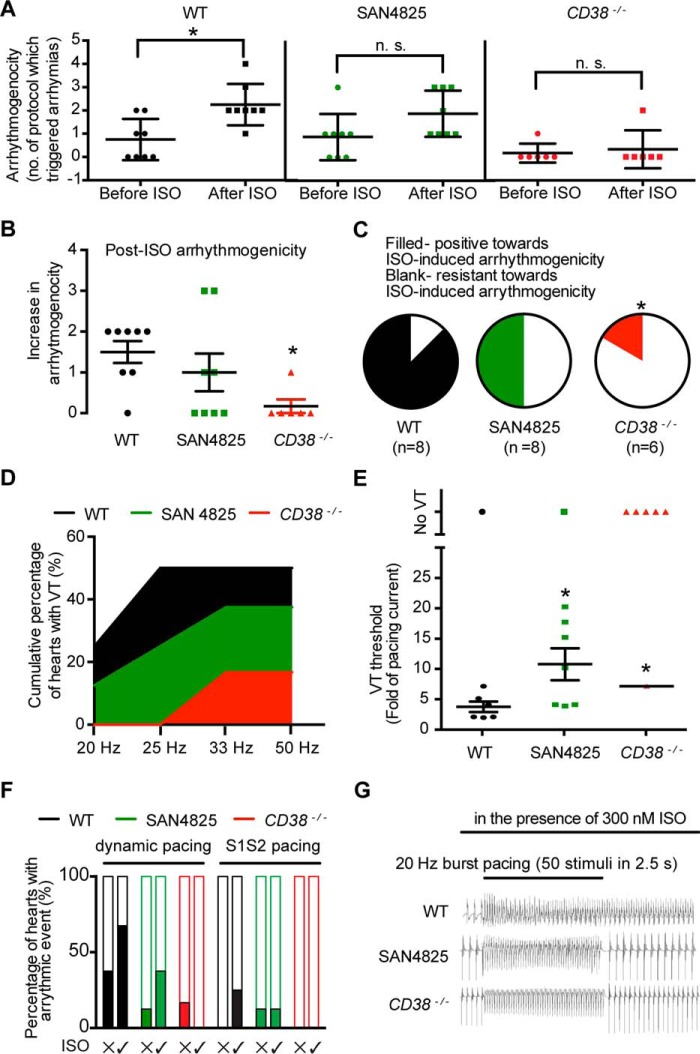
**Hearts from *CD38*^−/−^ mice or preincubated with SAN4825 showed resistance to arrhythmias during overstimulation of the β-adrenoreceptor pathway.** *A*, the tendency to show disturbances of rhythm was assessed using four different protocols (see “Experimental Procedures”) both before and after exposure to β-adrenoreceptor stimulation with isoproterenol (*ISO*, 300 nm). In WT hearts, exposure to isoproterenol caused the expected increase in arrhythmias, but this isoproterenol-induced tendency to arrhythmias was suppressed in hearts pretreated with SAN4825 as well as hearts from *CD38*^−/−^ mice. *B*, replotted from *A*, showing the increase in arrhythmogenicity of hearts in the presence of isoproterenol. *C*, the observations in *A* expressed as the proportion of hearts in which isoproterenol increased the tendency to arrhythmias. *D*, the cumulative percentage of hearts that exhibited arrhythmias in the presence of isoproterenol in the burst pacing experiments with increasing pacing frequency. *E*, the increase in threshold current required to induce ventricular tachycardia in the presence of isoproterenol. In experiments varying both pacing frequency and current amplitude, hearts from *CD38*^−/−^ mice and hearts preincubated with SAN4825 showed reduced tendency to arrhythmias in the presence of isoproterenol. *F*, the percentages of WT hearts, SAN4825-treated hearts, and *CD38*^−/−^ hearts that became arrhythmic (*filled columns*) during the dynamic pacing and S1S2 pacing experiments, with and without 300 nm isoproterenol. *G*, representative traces of burst pacing experiments. Data are expressed as mean ± S.E. or percentage. *A* and *B*, Mann Whitney Test. *C*, Fisher exact test. *E*, Student's *t* test or one-sample *t* test. *, *p* ≤ 0.05; **, *p* ≤ 0.01. WT, *n* = 8; SAN4825, *n* = 8; *CD38*^−/−^, *n* = 6; *n* = number of hearts.

It is known that excessive β-adrenoreceptor stimulation can lead to cardiac arrhythmias (41). The tendency to arrhythmias in intact mouse hearts was assessed by four different protocols (burst pacing with increasing frequency, 50-Hz burst pacing with increasing current, S1S2 pacing, and dynamic pacing; see “Experimental Procedures” for details and scoring methods). WT hearts showed a significant increase in arrhythmogenicity after application of 300 nm isoproterenol (Fig. 5*A*). In contrast, no significant increase in arrhythmogenicity was observed following isoproterenol application in hearts from *CD38*^−/−^ mice (Fig. 5*A*). Two other methods of data presentation were used to highlight changes in arrhythmogenicity. Fig. 5*B* shows the increase in arrhythmogenic events after isoproterenol and demonstrates the significant reduction in arrhythmogenicity in hearts from *CD38*^−/−^ mice compared with WT mice. When the observations were plotted as pie charts representing the proportion of hearts showing arrhythmias, it was clear that the proportion of hearts from *CD38*^−/−^ mice that were susceptible to isoproterenol-induced arrhythmogenicity (one of six hearts) was less than the corresponding proportion of WT hearts (seven of eight hearts, Fig 5C).

The cumulative percentage of hearts that became arrhythmic in the presence of isoproterenol (300 nm) during the burst pacing protocol with increasing pacing frequency and increasing pacing current is shown in Fig. 5*D*, again showing that hearts from *CD38*^−/−^ mice had a reduced tendency to arrhythmias under these conditions. Hearts from WT mice also had a significantly lower current threshold for initiation of arrhythmias, as shown in Fig. 5*E*. The percentage of *CD38*^−/−^ hearts that exhibited signs of arrhythmias (in the absence and presence of 300 nm isoprenaline) during dynamic pacing and S1S2 pacing protocols was also lower than those of WT hearts (Fig. 5*F*). Representative traces are shown in Fig. 5*G*. Taken together, these observations show that the tendency for arrhythmias during exposure to high concentrations of β-adrenoreceptor agonist was greatly reduced in hearts from *CD38*^−/−^ mice.

Having shown that 3 μm SAN4825 is sufficient to inhibit the production of NAADP and cADPR synthesis, we further investigated its anti-arrhythmogenic effect. The effects of 3 μm SAN4825 on the tendency for arrhythmias during excessive β-adrenoreceptor stimulation were broadly similar to the consequences of genetic knockdown of CD38 described above (Fig. 5, *A–G*), although the drug effects appeared to be less than CD38 knockout.

## Discussion

The main findings reported here are that CD38 is the principal enzyme in heart muscle responsible for synthesis of two Ca^2+^-mobilizing messengers, NAADP and cADPR, and that a major component of CD38 appears to be intracellular, perhaps associated with the SR (although there may be some additional CD38 enzyme on the cell surface). These findings are consistent with the conclusions of Gul *et al.* (15) that CD38 mediates synthesis of cADPR in cardiac myocytes but contrast with Gul *et al.* (15) in their proposal that a different enzyme (NAADP-synthesizing enzyme), is responsible for NAADP production in these cells.

When comparing our observations with those of Gul *et al.* (15), it is important to note differences in the experimental conditions, particularly with respect to the actions of isoproterenol. In our experiments, the effects of β-adrenoreceptor stimulation were studied under a physiological condition where cardiac myocytes were paced and the amplitude of CaTs triggered by electrical stimulation were investigated at low concentrations of isoproterenol (2–5 nm). In contrast, the increases in cytosolic Ca^2+^ reported by Gul *et al.* (15) were in cardiac myocytes that were not electrically paced and for which the isoproterenol concentration was 1000 times greater (2 μm). These very high concentrations of isoproterenol are likely to have caused spontaneous Ca^2+^ waves and/or oscillations, and the recorded maintained increases in cytosolic Ca^2+^ may have been the result of summation of signals (with an image collection rate of one per 3 s) from many cardiac myocytes in the field of view.

Gul *et al.* (15) have suggested that NAADP production precedes synthesis of cADPR and propose that Ca^2+^ released from lysosomes by NAADP reaches nearby CD38 in endosomes, causing an increase in the enzyme activity of CD38 to produce cADPR. This interpretation relies on observations in their Fig. 2, although it could be argued that the quite small observed differences in apparent time to peak synthesis of NAADP or cADPR (that appeared to be 15 s, or one measurement time point) may result from the ability to detect these messengers rather than reflect differences in the underlying physiology. Previous work in whole hearts showed that production of both cADPR and NAADP occurs rapidly following isoproterenol addition (11). It should also be mentioned that protein structural work shows that binding of Ca^2+^ causes conformational changes in CD38 that are expected to be inhibitory rather than stimulatory for enzyme action, whereas functional studies show little or no change in enzyme activity at the Ca^2+^ concentrations likely to be experienced by intracellular CD38 (42).

The possibility that the major active enzyme CD38 is located at or close to SR membranes is supported by our immunohistochemical observations showing that CD38 labeling has a striated pattern with a spacing that closely resembles that of the SR protein RyR2 both in ventricular and atrial myocytes. The lack of similar immunohistochemical staining in myocytes from *CD38*^−/−^ mice demonstrates that these observations are unlikely to result from lack of specificity of the antibody used to identify CD38. Because atrial myocytes lack the extensive t-tubule network seen in ventricular myocytes (and atrial t-tubules do not show the regular sarcomere spacing observed in the ventricle), the striated pattern also seen in atrial myocytes must result from staining of CD38 that appears to be associated with the SR (which does show a striated pattern in both atrial and ventricular muscle). It has recently been shown that lysosomes also show some association with the SR (as well as mitochondria), but the striated pattern for lysosomal markers is less distinct than the observations reported here for CD38 (36).

Although evidence for NAADP and cADPR function in cardiac myocytes has so far been demonstrated only in mice, rats, and guinea pigs (7–10, 43, 44) it is our hypothesis that this pathway operates in all mammalian hearts and that CD38 associated with the SR is the main synthetic enzyme. In the past, SR membrane preparations from the hearts of various animals have been used as a model to study the synthesis of cADPR or cGDPR by ARC (10, 14, 45, 46). In particular, sheep SR preparations have been widely used for the study of RyR2 and other SR proteins (27–34). We are the first to show that, in addition to catalysis of the cyclization reactions, sheep SR vesicles can also catalyze the base exchange reaction that produces NAADP (supplemental Fig. S4). Although the functional effects of this pathway have yet to be investigated in sheep, this species has the advantage of allowing easy preparation of amounts of biological material that are orders of magnitude greater than what can be prepared from much smaller mouse hearts. The relative purity of similar preparations is supported by electron microscopy and biochemical studies (45, 47, 48). Most importantly, to date, CD38 is the only mammalian enzyme that has been identified to have the ability to synthesize both cADPR and NAADP as a consequence of reactions at a multifunctional active site (see reviews in Ref. 49). Although we have yet to fully characterize the ARC on sheep SR, our initial investigations show that the enzymatic properties observed in sheep SR preparations have a high resemblance to those of human and mouse CD38. Therefore, our observations on sheep SR vesicles corroborate our hypothesis that CD38 is expressed intracellularly and appears to show a preferential association with the SR in cardiac tissue.

The functional observations with the Sanofi drug SAN4825, which reduced synthesis of both NAADP and cADPR, are consistent with its inhibitory actions at a single enzyme. The most favorable Glide docking conformations of SAN4825 with mouse CD38 at pH 7.2 suggest that this compound occupies the same binding site as the NAADP analogue and ADPRP, in close proximity with Glu-226. The pyridine ring of SAN4825 also shows π–π interaction with Trp-189 of mouse CD38. The blind docking results from the SwissDock server also predicted a single inhibitory binding site for SAN4825, and this site corresponds to the enzymatic active site of CD38. These SAN4285–CD38 binding conformations showed some resemblance to the native binding conformation between CD38 and its substrates, such as NAD, nicotinamide mononucleotide (NMN), cADRP, and NAADP (40, 50, 51).

Interestingly, the inhibition of NAADP but not cADPR synthesis by SAN4825 was observed to be greater at the cytosolic pH of 7.2 than at the acidic pH of 4.5 (Fig. 4, *A* and *B*). Therefore, we further investigated the binding conformations of SAN4825 at the active site of mouse CD38 with considerations of different protonation states of SAN4825 and mouse CD38 protein amino acids predicted by Epik and Propka 3.1, respectively (supplemental Fig. S6). The SAN4825 molecule can be protonated or unprotonated, and CD38 will adopt different protein conformations at different pH values. Nevertheless, with all four combinations (two SAN4825 protonation states and two CD38 conformations), all predicted binding sites of SAN4825 were still in proximity of the catalytic residues of mouse CD38 (supplemental Fig. S6). When considering the weakening of inhibition of NAADP synthesis by SAN4825 at pH 4.5, the following factors need to be taken into account: NAADP synthesis by human CD38 (3) and mouse CD38 (Fig. 4*A*) was observed to be enhanced at pH 4.5; inhibition of cADPR synthesis by SAN4825 remained unchanged at pH 4.5 (Fig. 4*B*); SAN4825 was predicted to bind to the CD38 active site at both pH 7.2 and pH 4.5 (supplemental Fig. S6); no NAADP synthesis was observed in membrane fractions from *CD38*^−/−^ mice at pH 4.5 (supplemental Fig. S7). In view of these observations and predictions, it appears that the observed weakened inhibition of SAN4825 at pH 4.5 does not result from a degradation of the drug or from a major change in the binding conformation of the drug and cannot be accounted for by a non-CD38 enzyme synthesizing NAADP. Although a weakened inhibition of NAADP synthesis by SAN4825 was observed at pH 4.5, evidence from our docking simulations and *in vitro* experiments still supports the ability of SAN4825 to inhibit the synthesis of NAADP and cADPR as a competitive inhibitor.

What are the implications of our findings for the normal physiology of the heart? Our observations show that the amplitudes of CaTs initiated by electrical stimulation at a constant rate were increased by β-adrenoreceptor stimulation with isoproterenol at low concentrations (2–5 nm) and that part of this response was prevented in WT mice by the use of bafilomycin A1 to suppress the contribution of the lysosomal pathway involving NAADP and TPC2 (9). In contrast, in myocytes from *CD38*^−/−^ mice, bafilomycin A1 no longer suppressed this component of the isoproterenol action, consistent with the importance of CD38 in the mechanism underlying NAADP production and the subsequent response. This is consistent with the need for coupling of CD38 to receptors, leading to NAADP synthesis and action via TPC2 proteins in the cardiac lysosomal signaling pathway (7, 44).

Previous experiments support elevation of NAADP and cADPR levels in response to β-adrenoreceptor stimulation by isoproterenol (7, 10, 11, 52). Our observations show that genetic knockout and pharmacological inhibition of CD38 provide resistance for arrhythmias associated with acute exposure to excessive stimulation with β-adrenoceptors compared with WT hearts. Taken together, these observations are consistent with an effect of β-adrenoreceptor stimulation by as yet unidentified mechanisms to enhance the catalytic activity of CD38, leading to increased levels of these Ca^2+^-mobilizing messengers (7, 11), which, in turn, enhance CaTs evoked by cardiac action potentials or trigger arrhythmic events resulting from excessive Ca^2+^-mobilizing messengers.

We have shown recently that the cardiac hypertrophy and associated tendency to arrhythmias that is provoked by chronic exposure to β-adrenoreceptor stimulation is markedly reduced when the influence of the NAADP pathway is suppressed in mice lacking NAADP-regulated TPC2 proteins (9). Because CD38 is upstream in this signaling pathway, it seems reasonable to propose that CD38 contributes to these processes and that hearts from *CD38*^−/−^ mice might also be resistant to cardiac hypertrophy and associated arrhythmias following chronic exposure to β-adrenoreceptor stimulation. The recent observations from Gul *et al.* (15) show that this is indeed the case and provide further support for this hypothesis.

Graeff *et al.* (53) have suggested that a protonation of NA at acidic pH is essential for the binding of NA to acidic residues (Glu-146 and Asp-155) on CD38 because, at neutral pH, the electrostatic repulsion between them would reduce substrate binding and, consequently, the rate of NAADP synthesis. However, our observations showed that cardiac muscle CD38 can catalyze the formation of NAADP at physiological pH, although at a lower rate than at acidic pH. It should also be noted that, as a result of the remarkably high potency of NAADP as a Ca^2+^ releasing messenger, the threshold concentration for NAADP to cause lysosomal Ca^2+^ release is very low, probably in the tens of nanomolar range (7), and, therefore, even a small amount of synthesized NAADP at pH 7.2 would be enough to activate the NAADP signaling pathway. This hypothesis is further supported by the work of Lee and co-workers (23), demonstrating that the active site of CD38 can remain functional under the reducing conditions of the cytosol and at cytosolic pH.

Although CD38 was thought to exist as a type II transmembrane protein with its C-terminal catalytic domain on the outside of the cell, Zhao *et al.* (22) showed, in cell lines and primary human cells, the presence of a proportion of CD38 in the type III form, with the catalytic domain facing the cytosol. In our study, substrates to permit synthesis of NAADP and/or cADPR were not included in the perfusion solution for intact heart or single-cell studies, and even if there was extracellular synthesis of these messengers, it seems likely that any relevant transporter of molecules will be made redundant or inefficient as a consequence of the constant flow of perfusion solution under the conditions of our experiments. These factors are expected to prevent the cytosolic accumulation of NAADP and cADPR from an extracellular source, and we have therefore favored arguments that, in cardiac cells, CD38 in the type III form might mediate intracellular synthesis of these Ca^2+^-mobilizing agents in WT type animals, leading to the observed effects on CaTs, contraction and arrhythmogenicity of the heart. This is consistent with our observations that permeabilized single cells (using saponin and/or Triton X-100) have higher enzymatic activity. Permeabilization was also required for immunolabeling of CD38. The finding that a functionally important component of CD38 appears to be intracellular and associated with the SR in the heart should not be considered to be in conflict with observations showing other locations for similar enzymes in different cell types (16, 19, 20, 54). It is possible that the Ca^2+^ signaling molecules cADPR and NAADP are so important in many diverse roles in the plant and animal kingdoms that different locations of synthetic enzymes may have evolved to suit different functional needs.

Because the known major modulatory influences of NAADP and cADPR on CaTs in cardiac myocytes have been shown to be mediated through SR-associated proteins (sarco-endoplasmic reticulum Ca^2+^-ATPase and RyRs) (9, 43, 55), it seems reasonable to propose that the most efficient operation of the signaling pathways involving NAADP and cADPR might be best served by a CD38 synthetic enzyme that is predominantly intracellular (perhaps located at or close to the SR membrane). An interesting hypothesis would be that CD38, NAADP, and cADPR as well as the relevant substrates could act within microdomains close to sites of Ca^2+^ uptake and/or release on the SR membrane, thereby facilitating the signaling pathways (see scheme in Ref. 44). Our recent observations concerning the location of lysosomes in close proximity to the SR are relevant in this context (36).

In the discussion above, the main focus is to highlight the pivotal role of intracellular CD38 in catalyzing the cyclization and base exchange reactions in hearts, particularly following activation of the β-adrenoreceptor pathway. There are several limitations to this study. Although we have proposed that the CD38 enzyme appears to be associated with SR membranes, we have not addressed the topology of the enzyme, particularly concerning the orientation of the active site, and this will be the subject of future experiments. In addition, although this study adds to previous work showing a link between β-adrenoreceptor stimulation and synthesis of NAADP and cADPR, further work is needed to establish the extent of activation of the CD38 pathway in the absence of this stimulation and to investigate the cellular mechanisms by which β-adrenoreceptor stimulation up-regulates CD38 activity.

In conclusion, this study provides novel evidence for cardiac synthesis of two potent and important Ca^2+^ mobilizing molecules, NAADP and cADPR, by intracellular CD38, probably associated with or close to the sarcoplasmic reticulum. The cellular pathway starting with CD38 and involving NAADP and cADPR acts to enhance excitation–contraction coupling, particularly during activation of β-adrenoceptors. Our observations demonstrating that *CD38*^−/−^ hearts are resistant to β-adrenoreceptor–associated arrhythmias also reveal the potential of targeting CD38-signaling pathways to treat cardiac disease.

## Experimental procedures

### Single-cell studies

#### 

##### Cell isolation

The *CD38*^−/−^ mouse line was developed by Cockayne *et al.* (56) and obtained from The Jackson Laboratory (*Cd38*^t m1L nd^/J). The protocol for generating and maintaining the mice was approved by the Oxford University Ethical Review Committee (Pharmacology Subcommittee) and Home Office. We confirm that all methods were performed in accordance with the relevant United Kingdom Home Office and institutional guidelines and regulations. Ventricular myocytes were isolated from male mice (C57BL6 or *CD38*^−/−^, 16–24 weeks old) and male New Zealand White rabbits (∼1 kg) using 0.3–0.5 mg ml^−1^ collagenase (type II, Worthington Biochemical Corp.) The detailed methods were described in Refs. 9, 36. Rabbit atrial myocytes were isolated using a similar protocol to that used to isolate rabbit ventricular myocytes.

##### Ca^2+^ *imaging in single cardiac myocytes*

Mouse myocytes were incubated with Fluo-5F/AM (5 μm) for 20 min. CaTs were stimulated at 1 Hz by carbon fiber electrodes placed at the side of the superfusion bath. Mouse ventricular myocytes were visualized using a Nikon Axiovert 200 inverted microscope with an attached Nipkow spinning disk confocal unit (CSU-10, Andor Technology). Excitation light was provided by a 488-nm diode laser (Cairn Research Ltd., Kent, UK) passed though the Nipkow unit and delivered to the sample through the objective. Emitted light passed back through the CSU-10 unit and was detected using an Andor iXON897 electron multiplying charge-coupled device (EMCCD) camera (Andor Technology) at 50 frames/s. Images were recorded and analyzed using Andor iQ software (Andor Technology). For analysis, background fluorescence was subtracted, and multiple transients were averaged to obtain the basal CaTs before isoproterenol application and the peak CaTs amplitude during 5 min of isoproterenol application. Data are presented as F/F_0_ so that fluorescence data are presented relative to diastolic fluorescence.

##### Contraction studies

Cells were stimulated at 1 Hz by carbon fiber electrodes placed at the side of the superfusion bath, and the contractile properties were studied using the IonOptix system (IonOptix Corp.) to measure sarcomere length. Cells were visualized via a ×40 oil objective using an IonOptix MyoCam (IonOptix Corp.) that sampled images at a frequency of 240 Hz. Sinusoidal optical density traces arising from the alternating light and dark bands of the contractile machinery were then transformed into a signal of sarcomere length by application of a fast Fourier transform by the IonWizard sarcomere length acquisition software (IonOptix Corp.). Length measurements were calibrated using a stage micrometer with 2-μm graduations so that the number of pixels per micrometer recorded by the MyoCam could be entered into the software as a fixed value. Amplitude of sarcomere shortening was calculated by deduction of systolic from diastolic sarcomere length. Analysis was performed using IonWizard 5 software (IonOptix Corp.). All values represent an average of 10 contractions. Cells for both Ca^2+^ imaging experiments and contraction studies were superfused with medium containing 130 mm NaCl, 5.4 mm KCl, 3.5 mm MgCl_2_, 1.8 mm CaCl_2_, 10 mm glucose, 5 mm HEPES, and 0.4 mm NaH_2_PO_4_ (pH 7.4) at 36 °C.

### In vitro biochemical assay

#### 

##### Mixed-membrane and sarcoplasmic reticulum–enriched membrane preparations

As described previously, mixed-membrane vesicles were prepared from sheep (obtained from an abattoir) or male mouse (C57BL6 or *CD38*^−/−^, 12–20 weeks old) cardiac tissue (57) A discontinuous sucrose gradient was used to separate the sarcolemmal membrane from the LSR and HSR membrane fractions. For each tissue, the protein concentration was calculated using a Bradford assay (58), and the various membrane fractions were aliquoted and stored at −80 °C.

##### Assessment of the enzymatic cyclization rate of ADP-ribosyl cyclase

A final concentration of 500 μm NGD was added to the protein sample to initiate the enzymatic reaction. cGDPR formation was monitored through its fluorescence intensity at 410 ± 5 nm during excitation, with light of a wavelength of 300 ± 3 nm (25). The cyclase activity of each reaction mixture was then defined by the change in fluorescence with time, *i.e.* the slope of the line in the fluorescence *versus* the time plot obtained by linear regression.

##### Detection and quantification of NAADP production

The level of NAADP production was quantified by means of an NAADP inactivation bioassay using SUEHs (24). The SUEHs were prepared using a method reported previously (59). Free Ca^2+^ concentration was measured with Fluo-3 by monitoring fluorescence intensity at excitation and emission wavelengths of 490 and 535 nm, respectively. To synthesize NAADP, 500 μm NADP and 7 mm NA were preincubated with the SR-enriched cardiac preparation at 37 °C. Then the protein–substrate mixtures were diluted before addition to the sea urchin egg homogenate. After 5 min, a standard 500 nm purified NAADP was further added to the sea urchin egg homogenate to elicit submaximal NAADP-induced Ca^2+^ release. The Ca^2+^ release response to the standard 500 nm NAADP is dependent on the subthreshold concentration of the NAADP in the preceding protein–substrate mixture addition. The concentration of NAADP in the protein–substrate mixture was quantified through interpolation of a standard dose-response curve from known concentrations of purified NAADP.

For both the NGD and SUEH assay, 10–20 μl of the SR-enriched or mixed-membrane cardiac preparation was incubated in a solution containing 150 mm KCl, 0.5 mm MgCl_2_, and 10 mm HEPES (pH 7.2). An acetate-based buffer was used in the condition where an acidic pH of 4.5 is required. For experiments investigating the enzymatic activities of permeabilized myocytes, myocytes were incubated with a solution comprising 70 mm KCl, 5 mm MgCl_2_, 5 mm potassium glutamine, 20 mm taurine, 0.04 mm EGTA, 5 mm succinic acid, 20 mm KH_2_PO_4_, 10 mm glucose, and 5 mm HEPES (pH 7.2) with KOH. This solution contained either 0.01% saponin (w/v) or 0.1% Triton X-100 (v/v). Fluorimetry was performed in a Novostar plate reader (BMG Labtech).

### Immunocytochemistry studies

Cells were fixed in in 4% paraformaldehyde/PBS for 15 min, washed in PBS (three changes, 5 min each), permeabilized using 0.1% Triton X-100 (Sigma-Aldrich) for 10 min, washed in PBS, and blocked with 1% bovine serum albumin for 60 min before being incubated with the primary antibody at 4 °C overnight. The next day, cells were first washed with PBS before being incubated with secondary antibody at room temperature for 2 h (either Alexa Fluor 488–conjugated donkey anti-rabbit or Dylight 550 donkey anti-mouse, 1:1000 dilution) and then washed. Finally, coverslips were mounted using Vectashield® and permanently sealed. Cells were stored in the dark at 4 °C and visualized within 2 days. For control experiments, we performed the same procedure with omission of the primary antibodies or with cells from *CD38*^−/−^ mice. Primary antibodies against CD38 (sc-15362, Santa Cruz Biotechnology) and RyR2 (ab2827, Abcam) were used at 1:100 and 1:200 dilution, respectively. Observations were carried out using a Nikon A1 confocal laser-scanning microscope equipped with a ×60 objective, and images were processed using ImageJ software.

### Ex vivo programmed electrical stimulations

Hearts were isolated from male mice (16–24 weeks old) and mounted in a Langendorff perfusion system. Hearts were perfused with Krebs solution comprising 119 mm NaCl, 25 mm NaHCO_3_, 4 mm KCl, 1.8 mm sodium pyruvate, 1.2 mm KH_2_PO_4_, 1 mm MgCl_2_, 1.8 mm CaCl_2_, and 10 mm glucose (pH 7.4) at 37 °C. Hearts were left to stabilize for 30 min. During this 30-min time window, hearts were also preincubated with drugs or vehicle present inside the Krebs solutions. To assess propensity to ventricular arrhythmias, hearts were subjected to four different programmed electrical stimulations in the absence and presence of 300 nm isoproterenol.

#### 

##### Frequency-varying burst pacing protocol

For the frequency-varying burst pacing protocol, a set of three trains of stimuli was delivered to the heart. Each train of stimuli consisted of 50 stimuli with intervals (cycle lengths) of 100 ms and was separated by a pacing-free interval of 2 s. The cycle length was progressively reduced from 100 to 20 ms in 10-ms decrements after each set.

##### Amplitude-varying burst pacing protocol

For the amplitude-varying burst pacing protocol, a set of three trains of stimuli was delivered to the heart. Each train of stimuli consisted of 50 stimuli with intervals of 20 ms and was separated by a pacing-free interval of 2 s. The current amplitude was progressively increased by factors of the pacing threshold current, *i.e.* the minimum current to evoke an action potential in a 1:1 manner. The protocol was terminated when a ventricular arrhythmia was observed or a current of 35 mA was reached.

##### Dynamic S1 pacing

Cycles of a decremental paced electrogram fractionation sequence comprising a 100-stimulus (S1) drive train were applied at a pacing interval of 135 ms initially. The pacing interval was reduced by 5 ms between successive drive trains until a pacing interval of 10 ms was reached.

##### S1S2 pacing

Cycles of a decremental paced electrogram fractionation sequence comprising an 8-Hz, eight-beat stimulus (S1) drive train, followed by an extra stimulus (S2), were applied. Initially, the interval between S1 and S2 was set to be 125 ms and was progressively reduced by 1 ms between successive drive trains until the preparation became refractory.

Ventricular arrhythmia was defined as six or more consecutive regular premature waveforms or irregular fibrillating waveforms. The arrhythmogenicity of the heart was determined by the number of protocols that successfully triggered arrhythmias. A scoring system was devised on this basis. For example, the score for arrhythmogenicity was 3 when a heart exhibited signs of arrhythmias under three of four protocols. The maximum score was 4 when all four protocols successfully triggered arrhythmias.

### In silico study

A conformational search in implicit solvent (water) with the OPLS_2005 force field (60) was performed for SAN4825 using MacroModel Schrödinger release 2017-1. An adapted blind docking of all possible conformations in the mouse CD38 (PDB code 2EG9, chain A) was performed with Glide (61, 62), considering all inside cavities, as determined by SiteMap (63). Protein was prepared using the protein preparation tool available in Maestro, filling in missing side chains and loops with Prime (64, 65) and minimizing the resulting structure with the OPLS_2005 force field. The protonation states of SAN4825 were predicted using the Epik software (66). Protein residue protonation states were determined by PropKa 3.1 (67, 68). Following docking, binding energies were calculated for ranking the best docking conformations using the molecular mechanics/generalized born surface area scoring function. For the scoring, residues close to 5 Å were considered flexible, allowing optimization of the docking conformations. To confirm that SAN4825 binds only to the active site containing the most important residues for NAADP and cADPR synthesis, SAN4825 was further submitted to a blind docking using the SwissDock server (69, 70). The distance between the predicted binding sites and the active site of CD38 was measured. The center of the active site of CD38 was determined to be the center of geometry of amino acid residues Trp-129, Glu-150, Asp-159, Trp-193, and Glu-230 of mouse CD38. These amino acid residues correspond to Trp-125, Glu-146, Asp-155, Trp-189, and Glu-226 in human CD38, which are important in CD38-mediated enzymatic syntheses, as identified in a site-directed mutagenesis study (38).

### Drugs and reagents

The SAN4825 compound was a kind gift from Sanofi (Montpellier, France). β-NGD, NADP, NA, nicotinamide, Triton X-100, saponin, and bafilomycin A1 were from Sigma (St. Louis, MO).

### Statistical analysis

All statistical comparisons were made by two-tailed Student's *t* tests unless stated otherwise. Mann-Whitney test and Fisher's exact test were used to compare arrhythmogenicity scores and categorical data, respectively. As only one in six hearts from *CD38*^−/−^ mice showed arrhythmias, one-sample *t* test (H_0_, μ = 7) was used for statistical analysis in Fig. 5*E*. All data are expressed as mean ± S.E.

## Author contributions

W. K. L. carried out all experiments, either solely or in collaboration with W. A. C. and M. P. J. (docking experiment), E. L. B. (contraction studies), Y. W. (MAP recording), F. O. B. (isolating SR fractions), and M. M. (sheep cardiac SR experiments). C. G. provided technical support for the plate reader experiments. M. R. was responsible for the breeding the CD38^−/−^ mouse line, and J. P. was the Home Office project license holder. W. K. L., A. G., M. L., and D. A. T. were responsible for the experimental design. All experiments were carried out in the laboratories of D. A. T., A. G., R. S., and M. L. All authors contributed to the writing and editing of the manuscript.

## Supplementary Material

Supplemental Data

## Footnotes

5 The abbreviations used are:

ARC: ADP-ribosyl cyclase
cADPR: cyclic ADP-ribose
NAADP: nicotinic acid adenine dinucleotide phosphate
SR: sarcoplasmic reticulum
SUEH: sea urchin egg homogenate
NGD: nicotinamide guanine dinucleotide
cGDPR: cyclic GDP-ribose
NA: nicotinic acid
HSR: heavy sarcoplasmic reticulum fraction
LHR: light sarcoplasmic reticulum fraction
CaT: Ca^2+^ transient
RyR: ryanodine receptor.
