# Supplementary material for: Synthesis of the Ca2+-mobilizing messengers NAADP and cADPR by intracellular CD38 enzyme in the mouse heart: Role in β-adrenoceptor signaling
Source: J Biol Chem. 2017 May 24;292(32):13243–57. doi: 10.1074/jbc.M117.789347 (PMC5555186; doi:10.1074/jbc.M117.789347)
Supplement: Supplemental Data [file supp_292_32_13243__index.html]

Synthesis of the Ca2+-mobilizing messengers, NAADP and cADPR, by intracellular CD38 enzyme in mouse heart: role in β-adrenoceptor signaling — Synthesis of the Ca2+-mobilizing messengers NAADP and cADPR by intracellular CD38 enzyme in the mouse heart: Role in β-adrenoceptor signaling — Intracellular CD38 in heart and β-adrenoceptor signaling — Supplemental Data 

# Synthesis of the Ca2+-mobilizing messengers NAADP and cADPR by intracellular CD38 enzyme in the mouse heart: Role in β-adrenoceptor signaling

## Supplemental Data

- supplementary (.pdf, 1.3 MB) - Supplementary figures
