# Supplementary material for: Synthesis of the Ca2+-mobilizing messengers NAADP and cADPR by intracellular CD38 enzyme in the mouse heart: Role in β-adrenoceptor signaling
Source: J Biol Chem. 2017 May 24;292(32):13243–57. doi: 10.1074/jbc.M117.789347 (PMC5555186; doi:10.1074/jbc.M117.789347)
Supplement: Supplemental Data [file 10.1074_M117.789347_jbc.M117.789347-1.pdf]

Synthesis of the Ca^{2+}-mobilizing messengers, NAADP and cADPR, by intracellular CD38 enzyme in mouse heart: role in β-adrenoceptor signaling

Wee K. Lin[1], Emma L. Bolton[1], Wilian A. Cortopassi[3,4], Yanwen Wang[2], Fiona O'Brien[1], Matylda Maciejewska[1], Matthew P. Jacobson[4], Clive Garnham[1], Margarida Ruas[1], John Parrington[1], Ming Lei[1], Rebecca Sitsapesan[1], Antony Galione[1], Derek A. Terrar[1].

From the [1]Department of Pharmacology, University of Oxford, Mansfield Road, Oxford, OX1 3QT, UK
[2]Faculty of Biology, Medicine and Health, University of Manchester, Manchester, M13 9NT, UK
[3]Department of Chemistry, Chemistry Research Laboratory, University of Oxford, Mansfield Road, Oxford, OX1 3TA, UK
[4]Department of Pharmaceutical Chemistry, University of California, San Francisco, California 94158, United States

Running title: *Intracellular CD38 in heart and β-adrenoceptor signaling*

To whom correspondence should be addressed: Prof Derek A Terrar, Department of Pharmacology, University of Oxford, Mansfield Road, Oxford OX1 3QT, UK. Telephone: (+44) 1865271943; FAX: (+44) 1865271853; E-mail: derek.terrar@pharm.ox.ac.uk

Keywords: Ca^{2+}, beta-adrenoceptor, NAADP, cADPR, CD38, lysosomes, sarcoplasmic reticulum, heart, cardiac arrhythmia, cardiac hypertrophy

---

List of figures:

Figure S1: Effects of H_20, Triton X-100, saponin and DMSO on the sea urchin egg homogenates bioassay

Figure S2: Alignment of sequence of human CD38 and mouse CD38

Figure S3: Immunolabeling of CD38 on rabbit atrial myocytes

Figure S4: Sheep cardiac SR showed ADP-ribosyl cyclase activities and NAADP production with properties similar to those of CD38

Figure S5: SAN4825 inhibited the production of NAADP in saponin-permeabilized single ventricular myocytes from mouse hearts

Figure S6: Binding conformations of SAN4825 at different pH

Figure S7: NAADP synthesis at pH 4.5 in cardiac membrane preparation from *CD38*$^{-/-}$ mice

REFERENCES

1. Altschul, S. F., Gish, W., Miller, W., Myers, E. W., and Lipman, D. J. (1990) Basic local alignment search tool., *J. molecular biology* **215**(3), 403–10. 10.1016/S0022-2836(05)80360-2
2. Altschul, S. F., Madden, T. L., Schäffer, A. A., Zhang, J., Zhang, Z., Miller, W., and Lipman, D. J. (1997) Gapped BLAST and PSI-BLAST: A new generation of protein database search programs
3. Munshi, C., Aarhus, R., Graeff, R., Walseth, T. F., Levitt, D., and Lee, H. C. (2000) Identification of the enzymatic active site of CD38 by site-directed mutagenesis, *J. Biol. Chem.* **275**(28), 21566–21571. 10.1074/jbc.M909365199

FIGURES

Figure S1

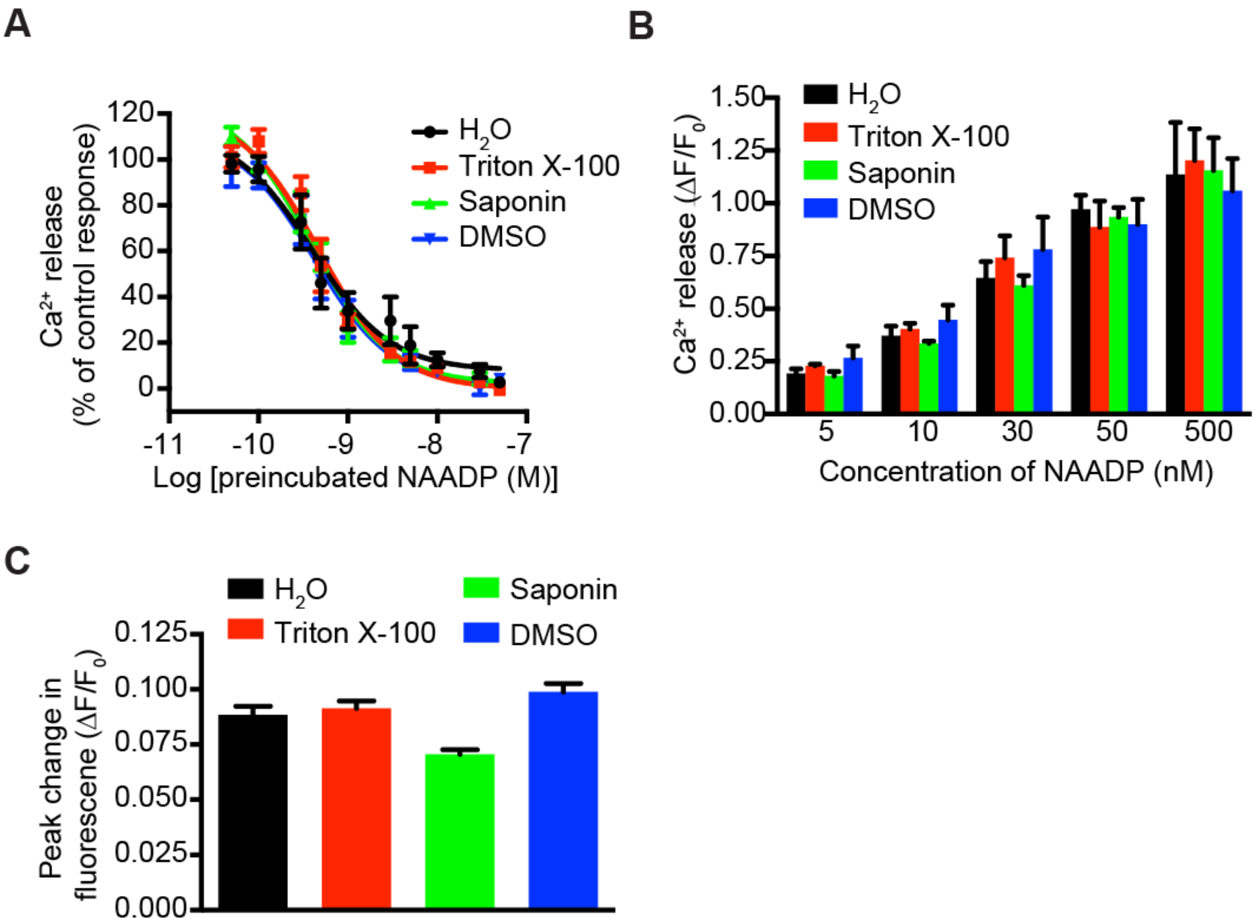

Figure S1: Effects of H_20, Triton X-100, saponin and DMSO on the sea urchin egg homogenates bioassay

A shows the homologous inactivation properties of NAADP in the SUEH, while **B** shows the ability of NAADP to mediate Ca^{2+} release in SUEH in the presence of H_20, Triton X-100, saponin or DMSO. **C** shows the change in Ca^{2+}-dependent fluorescence after the addition of H_20, Triton X-100, saponin or DMSO in the SUEH bioassay. Note that all addition artifacts are relatively small (< 10 %) compared to the change in Ca^{2+}-dependent fluorescence caused by 500 nM NAADP. These control experiments showed that neither the homologous inactivation properties nor the NAADP-mediated Ca^{2+} release was affected by permeabilizing agents or solvents used in our studies, supporting the reliability of NAADP measurement using this assay system under the conditions of our experiments. The concentrations of Triton X-100 (5 x 10^{-5} % v/v), saponin (5 x 10^{-6} % w/v) and DMSO (5 x 10^{-4} % v/v) that were used in these experiments correspond to the highest final concentrations present in SUEH in our studies. Data are expressed as the mean ± SEM; n = 3 independent experiments.

Figure S2

```
MANYEFSQVSGDRPGCRLSRKAQIGLGVGLLVLIALVVGIVVILLRPRSLL51  mouse
MANCEFSPVSGDKPCCRLSRRAQLCLGVSILVLILVVVLAVVV---PRWRQ48  human

VWTGEPTTKHFSDIFLGRCLIYTQILRPEMRDQNCQEILSTFKGAFVSKNP102 mouse
QWSGPGTTKRFPETVLARCVKYTEI-HPEMRHVDCQSVWDAFKGAFISKHP98  human

CNITREDYAPLVKLVTQTIPCNKTLFWSKSKHLAHQYTWIQGKMFTLEDTL153 mouse
CNITEEDYQPLMKLGTQTVPCNKILLWSRIKDLAHQFTQVQRDMFTLEDTL149 human

LGYIADDLRWCGDPSTSDMNYVSCPHWSENCPNNPITVFWKVISQKFAEDA204 mouse
LGYLADDLTWCGEFNTSKINYQSCPDWRKDCSNNPVSVFWKTVSRRFAEAA200 human

CGVVQVMLNGSLREPFYKNSTFGSVEVFSLDPNKVHKLQAWVMHDIEGASS255 mouse
CDVVHVMLNGSRSKIFDKNSTFGSVEVHNLQPEKVQTLEAWVIHGGREDSR251 human

NACSSSSLNELKMIVQKRNMIFACVDNYRPARFLQCVKNPEHPSC      300 mouse
DLCQDPTIKELESIISKRNIQFSCKNIYRPDKFLQCVKNPEDSSC      296 human
```

Figure S2: Alignment of sequence of human CD38 and mouse CD38
The region used to raise the antibody was highlighted in yellow. The sequences were aligned using protein-protein Blast (1, 2) and the identical amino acid residues from mouse and human CD38 were labeled with red fonts. The amino acid residues surrounded by green boxes are those shown to be critical for the human CD38 enzymatic activities, determined by a site-mutagenesis study (3). The mouse catalytic residues corresponding to the Trp125, Glu146, Asp155, Trp189 and Glu226 of human CD38 are Trp129, Glu150, Asp159, Trp193 and Glu230, respectively. All catalytic residues are conserved between mouse and human CD38.

Figure S3

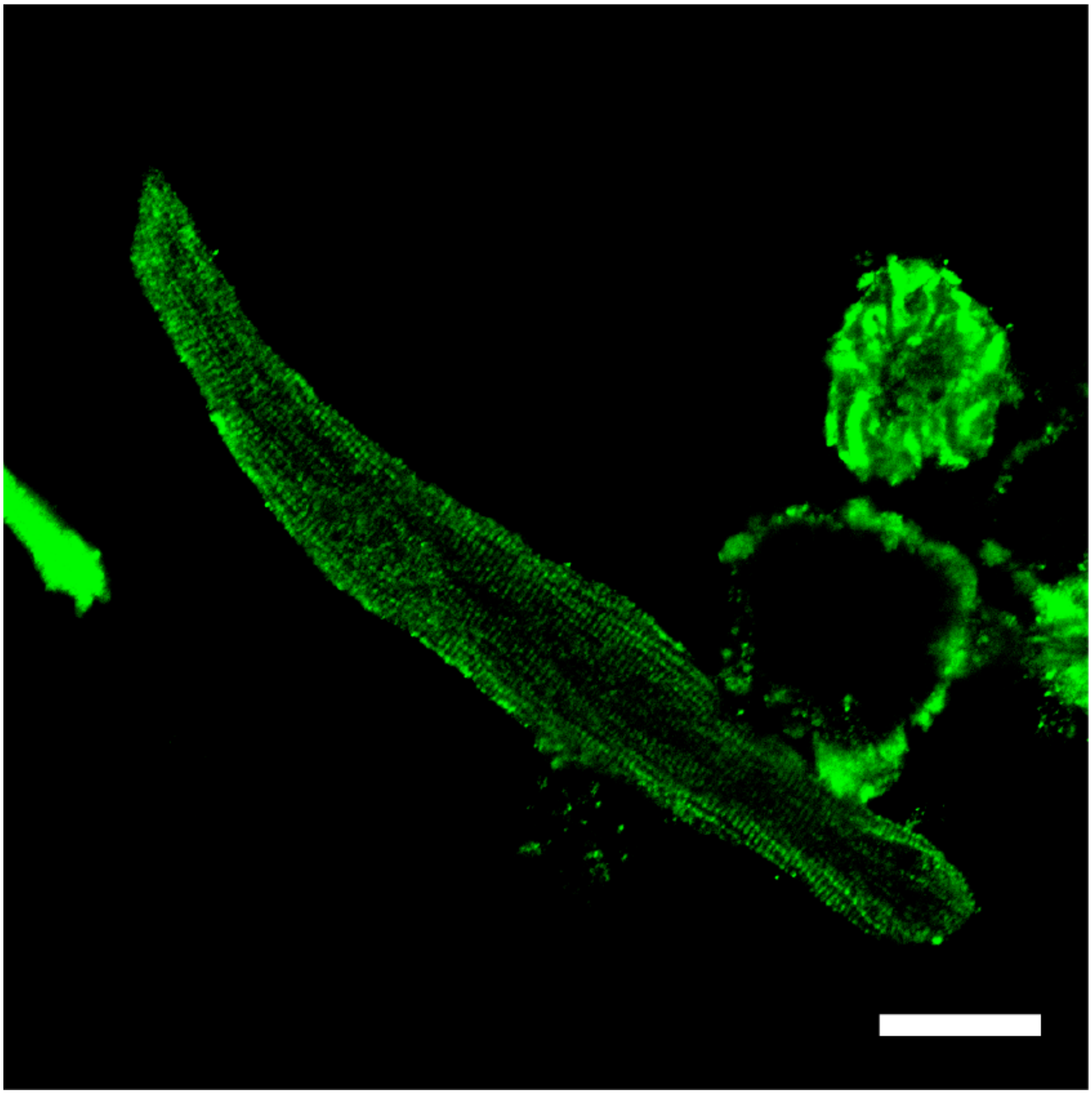

Figure S3: Immunolabeling of CD38 on rabbit atrial myocytes
Figure S3 shows the immunolabeling of CD38 with a striated pattern corresponding to the location of SR (and not that of surface membrane). Images shown are the representative staining of the majority (> 75%) observations (number of cells observed >20 isolated from 2 rabbit hearts). Antibodies used: rabbit polyclonal anti-CD38 antibody (sc-15362); scale bar: 20 μm.

Figure S4

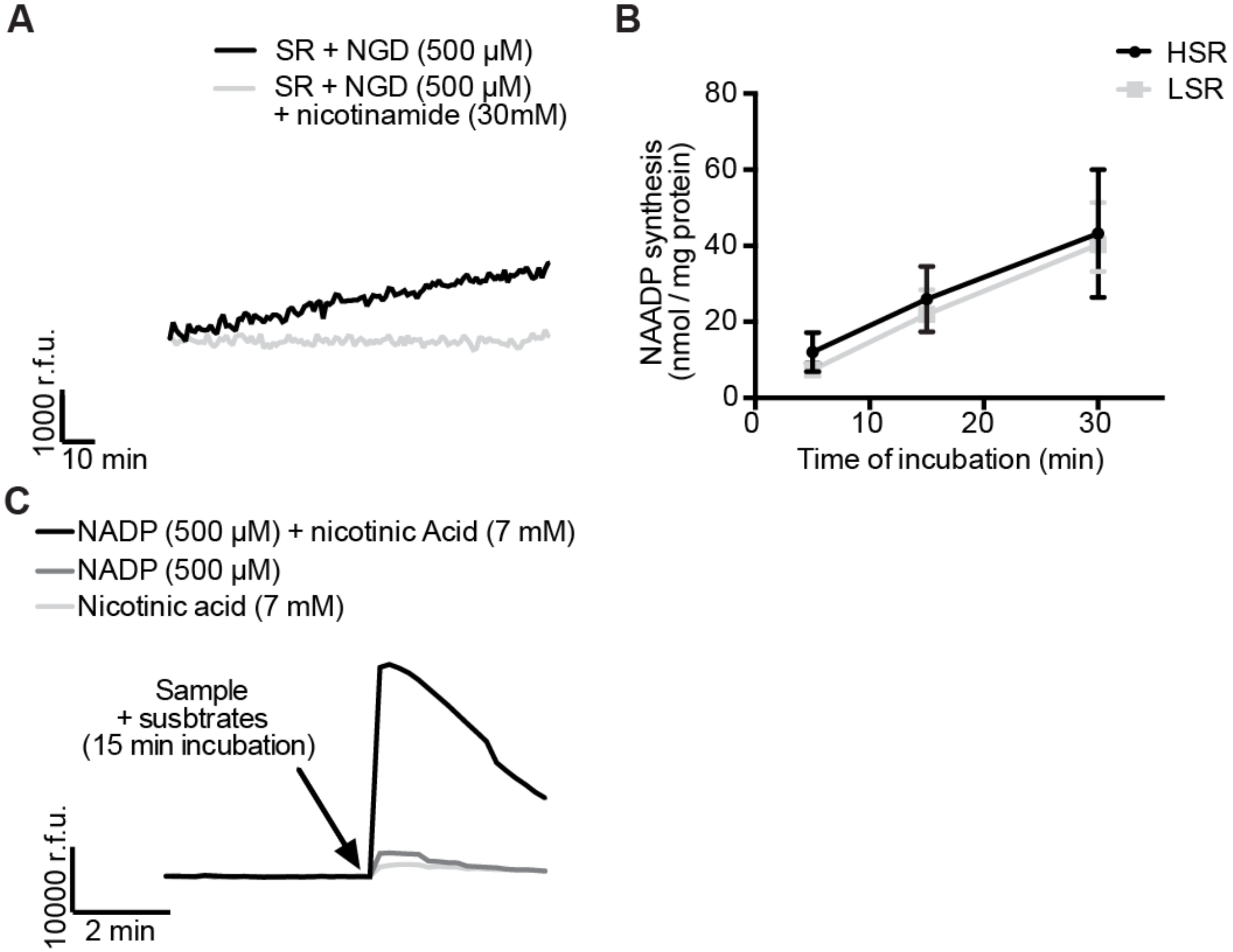

Figure S4: Sheep cardiac SR showed ADP-ribosyl cyclase activities and NAADP production with properties similar to those of CD38

A shows an example trace of synthesis of fluorescent cGDPR by sheep cardiac SR with NGD as substrate. This synthesis was suppressed in the presence of a CD38 inhibitor, nicotinamide. **B** shows the time course of NAADP synthesis in preparations enriched in heavy or light SR (similar in both preparations; $p = 0.9226$). **C** shows an example trace of NAADP synthesis with NADP as a substrate in the presence of NA, while synthesis was suppressed with either NADP or NA alone. Data in B are expressed as the mean ± SEM; $n \geq 3$ independent experiments.

Figure S5

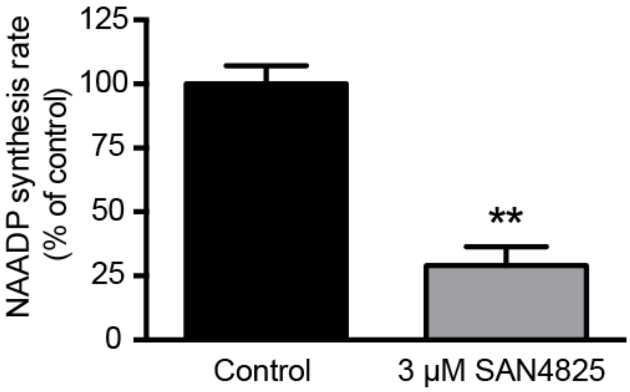

Figure S5: SAN4825 inhibited the production of NAADP in saponin-permeabilized single ventricular myocytes from mouse hearts

Figure S5 shows that the NAADP synthesis rate in saponin-permeabilized single ventricular myocytes from mouse hearts was significantly reduced in the presence of 3 μM SAN4825. Both control and SAN4825-treated myocytes were first permeabilized with saponin (0.01 % w/v), preincubated with vehicle or drug for 30 min before being supplemented with 500 μM NADP and 7 mM NA to initiate the NAADP production. Data are expressed as the mean ± SEM; ** $p < 0.01$ relative to control, determined by Student's t-test. $n = 4$ independent experiments.

Figure S6

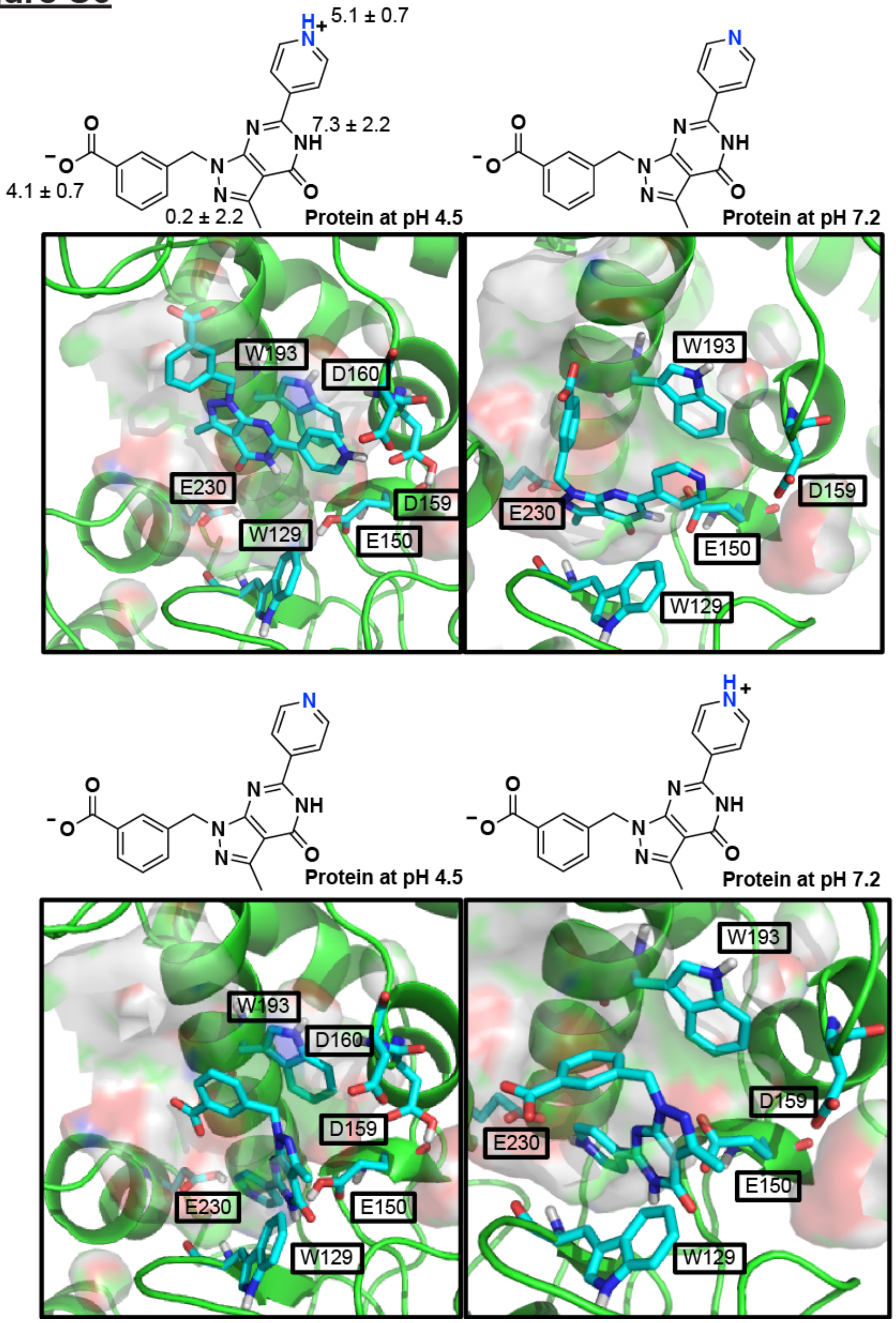

Figure S6: Binding conformations of SAN4825 at different pH

The four images, each showing a SAN4825-CD38 complex, represent the predicted binding conformation of SAN4825 at the active site of mouse CD38 (PDB ID: 2EG9) under different conditions. The predictions were performed with consideration of the influence of pH on the protonation states of SAN4825 and on the amino acids of the CD38 protein, which were predicted by Epik and Propka 3.1, respectively. The predicted pKa values for SAN4825 are shown in top left. The protonation of the nitrogen (highlighted in blue) of the pyridine with a pKa value of 5.1 is likely to change in the pH range of 4.5 to 7.2. The term "protein at pH 4.5" refers to a mouse CD38 protein with protonated states predicted at pH 4.5 while the term "protein at pH 7.2" refers to a mouse CD38 protein with protonated states predicted at pH 7.2. The chemical structure above each image depicts the protonation state of SAN4825 considered.

Figure S7

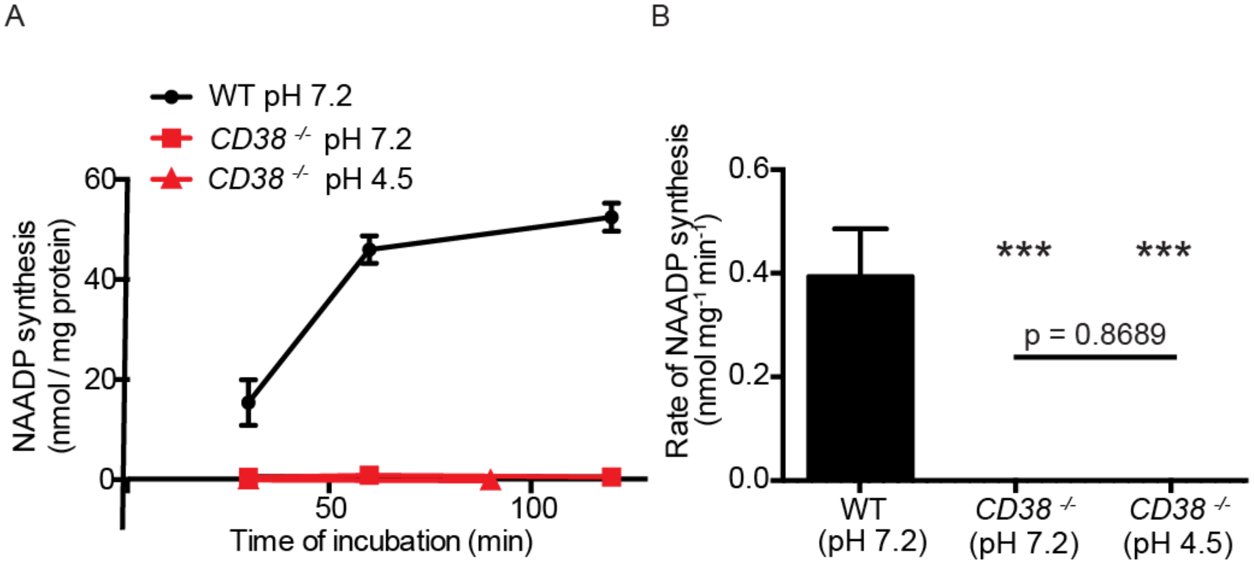

Figure S7: Lack of *In vitro* NAADP synthesis at pH 4.5 in cardiac membrane preparation from *CD38*$^{-/-}$ mice

A and **B** show the time course and average rate of NAADP synthesis by cardiac membrane preparation from WT and *CD38*$^{-/-}$ mice at different pH. The NAADP synthesis of membrane preparation from *CD38*$^{-/-}$ mice remained reduced and comparable with those of *CD38*$^{-/-}$ mice at pH 7.2. Data are expressed as the mean ± SEM; ** $p < 0.01$ relative to control, determined by Student's t-test. n = 3 independent experiments.
